# Managing Cough in Pediatric Neuromuscular Disorders: Lung Function and Care Strategies

**DOI:** 10.3390/children12101377

**Published:** 2025-10-12

**Authors:** Simone Foti Randazzese, Grazia Fenu, Claudia Calogero, Enrico Lombardi, Sara Manti

**Affiliations:** 1 Pediatric Unit, Department of Human Pathology in Adult and Developmental Age “Gaetano Barresi”, University of Messina, Via Consolare Valeria, 1, 98124 Messina, Italy; smanti@unime.it; 2 Pediatric Pulmonary Unit, Meyer Children’s Hospital, IRCCS, Viale Pieraccini, 24, 50139 Florence, Italy; grazia.fenu@meyer.it (G.F.); claudia.calogero@meyer.it (C.C.); enrico.lombardi@meyer.it (E.L.)

**Keywords:** cough, neuromuscular disorders, lung function, care strategies, pediatrics

## Abstract

Children with neuromuscular disorders (NMDs) are at high risk for respiratory complications due to impaired cough and weakness of respiratory muscles. Effective cough is essential for airway clearance, infections’ prevention, and maintaining lung function. This narrative review explores the physiology of cough, the consequences of cough insufficiency, and methods for assessing respiratory function in pediatric NMDs. It discusses current care strategies including airway clearance techniques, respiratory muscle training, and preventive and supportive interventions. Emphasis is placed on multidisciplinary management and early intervention to improve outcomes and quality of life.

## 1. Introduction

Neuromuscular disorders (NMDs) encompass a broad group of conditions characterized by dysfunction of the motor unit, which includes the anterior horn cells, peripheral nerves, neuromuscular junctions, and skeletal muscles [1].

In children, most NMDs are genetically determined, resulting from inherited or de novo pathogenic variants in single genes. These include dystrophinopathies (e.g., Duchenne and Becker muscular dystrophies); spinal muscular atrophy (SMA), a motor neuron disease affecting the anterior horn cells; myotonic dystrophy, a multisystemic disorder with prominent myopathic features; Charcot–Marie–Tooth disease (CMT), a hereditary neuropathy involving peripheral nerves; metabolic myopathies, such as Pompe disease, a glycogen storage disorder with cardiomyopathy; and mitochondrial myopathies, resulting from defects in oxidative phosphorylation, frequently accompanied by neurological and systemic symptoms [2,3,4].

In contrast, acquired NMDs such as myasthenia gravis, Guillain–Barré syndrome, and chronic inflammatory demyelinating polyneuropathy are typically immune-mediated [5].

Although individually rare, their collective prevalence is significant, affecting approximately 1 in 3000 children [6,7].

Clinically, NMDs can manifest with different chronic symptoms such as progressive muscle weakness, impaired gait and ambulation, joint contractures, skeletal deformities (e.g., scoliosis), and in neuropathic subtypes, altered sensory perception. Additionally, patients may experience episodic or fluctuating symptoms, including exercise intolerance, myalgia, rhabdomyolysis, and fatigable weakness, sometimes in the context of preserved baseline function [2,8].

Respiratory system compromise is a major concern in NMDs and may result from a combination of factors, including respiratory muscle weakness, bulbar hypotonia (as seen in lower motor neuron disorders or myopathies), bulbar hypertonia (as seen in upper motor neuron disorders), scoliosis, and impaired central respiratory drive. These factors reduce airway tone, impair cough efficiency and secretion clearance, and alter chest wall mechanics, making respiratory dysfunction a potentially life-threatening complication [9,10].

Proactive and systematic evaluation is essential because respiratory symptoms may be subtle, especially in those patients who are seen in the outpatient clinic. The early identification and management of cough insufficiency are particularly critical, as ineffective airway clearance significantly increases the risk of infection and morbidity [11].

This narrative review aims to provide a comprehensive overview of cough insufficiency in pediatric NMDs, focusing on the pathophysiology, respiratory assessment, clinical implications of impaired cough, and evidence-based management strategies. Emphasis is placed on multidisciplinary care and early intervention to improve long-term outcomes and quality of life (QoL).

A literature search was conducted via PubMed using the following key terms: “pediatric neuromuscular disorders” OR “neuromuscular diseases” AND “respiratory compromise” OR “respiratory complications” AND “cough insufficiency” OR “ineffective cough” AND “respiratory function” OR “lung function” AND “airway clearance” AND “mechanical insufflation-exsufflation” AND “care strategies” AND “children” OR “pediatrics.” Articles published between 2000 and 2025, including original studies, reviews, systematic reviews, meta-analyses, randomized controlled trials (RCTs), observational studies, and clinical guidelines, were considered. Additional sources were identified through manual review of reference lists.

Studies were selected based on their relevance to pediatric NMDs and their focus on cough-related respiratory dysfunction. Given the heterogeneity of study designs, populations, and outcome measures, a formal meta-analysis was not feasible. Instead, a narrative synthesis was undertaken. We acknowledge that this approach limits the ability to quantify effect sizes or assess statistical rigor across studies; however, it allows for a broader integration of findings across diverse methodologies and clinical contexts.

## 2. Cough Mechanics and Dysfunction in Neuromuscular Disorders

Cough is a vital respiratory defence mechanism defined as a forceful expiratory effort against a closed glottis, producing a characteristic sound. It is a protective reflex to expel mucus, aspirated material, and inhaled irritants from the airways [12].

### 2.1. Neurophysiology and Biomechanics of Cough

Cough may be voluntary or reflexive and it can be triggered by mechanical or chemical stimuli such as mucus, airway inflammation, cold air, or smoke [13].

Sensory detection relies on various ion channels and receptors, particularly P2X3, acid-sensing ion channels, and members of the transient receptor potential (TRP) superfamily, including TRP Vanilloid 1 (TRPV1), TRP Ankyrin 1 (TRPA1), TRP Vanilloid 4 (TRPV4) and TRP Melastatin 8 (TRPM8), located on vagal afferents in the larynx, trachea, bronchi, and extra-respiratory sites such as the pharynx, sinuses, pleura, and diaphragm [14,15,16,17]. Upon stimulation, sensory input is transmitted via the vagus nerve to the brainstem in the medulla oblongata, where the cough reflex is processed. From the brainstem, efferent signals are then sent via the vagus, phrenic, and spinal nerves to coordinate the muscular actions required to produce an effective cough [18].

Specifically, the cough reflex proceeds through a well-defined sequence of motor events. During the inspiratory phase, a deep breath (~50% of vital capacity) increases lung volume, thereby optimizing the conditions for subsequent forceful airflow. This is followed by the compressive phase, involving rapid closure of the glottis for 0.2 s and simultaneous contraction of the expiratory muscles, particularly the abdominal and intercostal muscles, to generate an intrathoracic pressure greater than 200 cmH_2_O. The process culminates in the expiratory phase, marked by a sudden opening of the glottis while the expiratory muscles continue to contract and the rapid release of pressurized air with the production of a cough peak flow (CPF) greater than 400 L/min that expels mucus and irritants [19,20].

### 2.2. Mechanisms of Cough Impairment

In children with NMDs, progressive respiratory muscle weakness compromises the cough reflex’s biomechanical integrity. Effective cough depends on generating high expiratory airflow, and dysfunction in any component of the cough pathway leads to impaired airway clearance and increased susceptibility to several complications that can go from recurrent respiratory tract infections (RTIs), to atelectasis, to respiratory failure [21,22].

The pathophysiology underlying ineffective cough in NMDs involves disruption across multiple phases of the cough reflex arc. Inspiratory muscle weakness, particularly involving the diaphragm, reduces the capacity for adequate pre-cough lung inflation, limiting the volume required for sufficient elastic recoil. This impairment is often compounded by restrictive thoracic deformities (e.g., scoliosis). During the compressive phase, abdominal and intercostal muscle weakness reduces intrathoracic pressure, while bulbar dysfunction interferes with proper glottic closure. In the expiratory phase, impaired glottic coordination and weak expiratory muscles result in reduced airflow velocity [22].

### 2.3. Microbial Colonization and Respiratory Morbidity

Ineffective cough leads to impaired airway clearance with stasis and retained secretions, promoting microbial colonization. The mucociliary system, composed of ciliated epithelial and secretory cells, serves as a primary defence mechanism by facilitating the removal of inhaled particles and pathogens [23,24]. Secretory cells (e.g., goblet cells, submucosal glands, and Clara cells) release a variety of antimicrobial and immunomodulatory molecules, such as lysozyme, lactoferrin, nitric oxide, and proteases, embedded within a mucus matrix formed by gel-forming mucins (MUC5AC and MUC5B) that trap pathogens for clearance [25,26].

In children with NMDs and ineffective cough, retained secretions provide a favourable substrate for colonization by pathogens such as *Pseudomonas aeruginosa*, *Haemophilus influenzae*, and *Staphylococcus aureus* [25,26]. For instance, *P. aeruginosa* impairs mucociliary function by secreting proteases that damage epithelial cells and reduce ciliary beat frequency [27,28,29]. Persistent colonization drives chronic neutrophilic inflammation, mediated by chemokines and cytokines, including interleukin-8 (IL-8), IL-1β, tumour necrosis factor-α, and leukotriene B4 [30]. Although aimed at microbial clearance, this response paradoxically impairs neutrophil function, causing further epithelial damage and mucus stasis [31,32]. This leads to a self-perpetuating vicious cycle of secretion retention, infection, and inflammation, which contributes to recurrent pulmonary exacerbations commonly associated with hypoxemia, increased respiratory effort, and segmental or lobar atelectasis [33].

A retrospective cross-sectional study involving 77 children with NMDs (mean age 15.6 ± 5.4 years) documented that 51% of participants presented upper airway microbial colonization, which was significantly associated with poorer respiratory outcomes. Colonized patients exhibited lower forced vital capacity (FVC) and CPF than non-colonized peers (FVC: 26.6 ± 19.7% vs. 41.8 ± 20.4%, *p* < 0.001; CPF: 125 ± 55 L/min vs. 207 ± 100 L/min, *p* < 0.001). Colonization with Gram-negative bacteria or *S. aureus* further worsened these parameters (FVC: 22.6 ± 16.5% vs. 37.9 ± 21.5%, *p* = 0.003; CPF: 123 ± 60 L/min vs. 179 ± 93 L/min, *p* = 0.013). These findings highlight the clinical impact of microbial colonization in children with NMDs and the importance of routine microbiological monitoring to guide preventive respiratory care [34].

**Figure 1:**
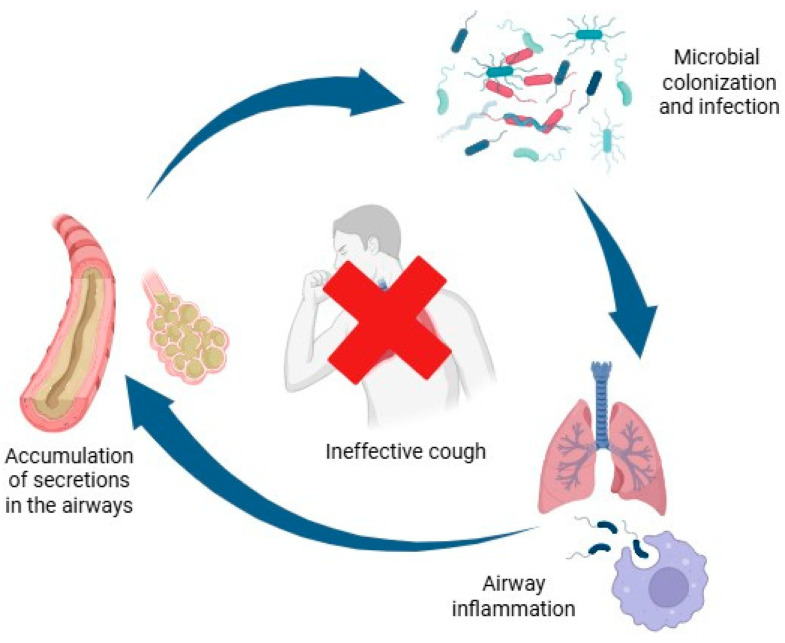
Respiratory complications of ineffective cough in NMDs.

Figure 1 summarizes the respiratory complications associated with ineffective cough in children with NMDs.

## 3. Assessment of Lung Function and Cough Efficacy

**Figure 2:**
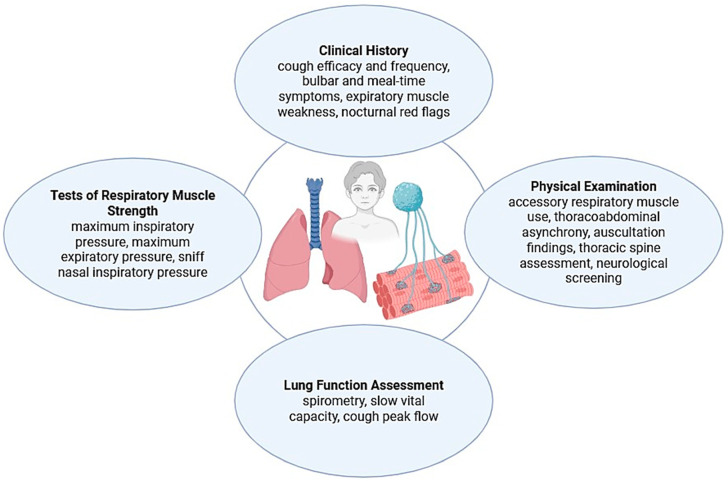
Key clinical signs and functional assessments of cough insufficiency in children with NMDs.

In children with NMDs, cough insufficiency is a marker of disease progression as it can be one of the first signs of respiratory involvement and a major contributor to respiratory morbidity, hospitalizations, and infections. Routine evaluation of cough function is crucial [22]. Figure 2 summarizes the key clinical signs and functional assessments essential for early detection and monitoring of cough dysfunction.

### 3.1. Clinical Evaluation

Clinical history and physical examination play a critical role in identifying early signs of cough dysfunction, enabling timely intervention [7,35].

A thorough clinical history should assess cough efficacy and frequency, especially during RTIs, as well as symptoms of bulbar involvement, such as nasal regurgitation and dysphagia involving liquids or solids, which are common in pharyngeal muscle weakness and may indicate silent aspiration—an underrecognized cause of respiratory complications [7,34,36,37]. Coughing or choking during meals may reflect impaired airway protection, while absent voluntary cough may suggest a combination of glottic insufficiency and expiratory muscle weakness [38]. Nocturnal symptoms—morning headaches, non-restorative sleep, excessive daytime sleepiness, enuresis, and orthopnea—often signal early diaphragmatic dysfunction and nocturnal hypoventilation [7,20,38,39].

Physical examination provides key objective findings on using accessory respiratory muscles, thoracoabdominal asynchrony, and paradoxical breathing during inspiration are classic signs of respiratory muscle weakness [35,38,40,41]. Auscultation may reveal diminished breath sounds due to low tidal volumes or crackles from atelectasis and impaired mucociliary clearance [20,38,42]. The examination of the thoracic spine is also essential, as scoliosis can worsen restrictive lung patterns [7,43].

Neurological assessment should screen for bulbar dysfunction—manifested by dysphagia, sialorrhea, and reduced cough reflex—which increases aspiration risk [36,37,42].

### 3.2. Lung Function Assessment

Objective pulmonary function testing (PFT) is essential for monitoring respiratory status and guiding management in children with NMDs [38,44].

#### 3.2.1. Spirometry

Spirometry remains the gold standard in PFT, suitable for children over six years of age who can perform reproducible efforts [45].

In progressive NMDs such as Duchenne Muscular Dystrophy (DMD) or SMA (types 2 and 3), PFT is recommended every six months, even in the absence of overt respiratory symptoms. During clinical deterioration, assessments may be required every 3–6 months. In contrast, children with non-progressive or milder forms may undergo annual assessments [44].

Spirometric values are expressed in absolute terms and percent predicted based on age, sex, ethnicity, and height [45]. However, in patients with skeletal deformities, surrogate anthropometric markers such as arm span or ulna length should be used [38]. Additionally, mouthpiece challenges from facial or bulbar weakness can be addressed using flanged mouthpieces or face masks [38].

Typical spirometric patterns in NMDs include reduced FVC and forced expiratory volume in the 1st second (FEV_1_), with a preserved or elevated FEV_1_/FVC ratio (80–100%), indicative of a restrictive pattern [35,46].

Recent American Thoracic Society/European Respiratory Society technical standards on interpretive strategies for routine lung function test recommend interpreting results using z-scores instead of fixed percentage thresholds. A z-score between −1.65 standard deviation (SD) and −2.5 SD indicates a mild deficit, between −2.51 SD and −4.0 SD a moderate deficit, and a z-score below −4.1 SD indicates a severe reduction in lung function [45].

Beyond its pulmonary applications, spirometry correlates well with upper and lower extremity skeletal muscle functional testing such as accelerometry and quantitative muscle testing (QMT). Recently, a prospective observational study explored this relationship in 35 patients (mean age 14.2 ± 3.9 years) with DMD. FVC and FEV_1_ demonstrated the strongest correlations with accelerometry, particularly within low-intensity and moderate-to-vigorous physical activity categories. This suggests that declining lung function is closely aligned with reducing overall activity levels. Additionally, maximal expiratory pressure (MEP) and FVC showed robust associations with total QMT scores, with statistically significant correlations (*p* < 0.001 and *p* < 0.01, respectively) [47].

#### 3.2.2. Slow Vital Capacity

Due to muscle weakness, some children may be unable to perform forced expiratory manoeuvers reliably. The measurement of slow VC (SVC), which involves maximal inhalation followed by slow, complete exhalation, is generally more feasible and offers valuable information on inspiratory muscle strength and chest wall compliance. SVC should be routinely measured, particularly in the upright position to optimize diaphragmatic descent. However, supine SVC measurements may be more sensitive for detecting diaphragmatic weakness and tracking disease progression [35]. A ≥25% drop from upright to supine positions is highly sensitive (90%) and specific (79%) for diaphragmatic dysfunction [48].

#### 3.2.3. Cough Effectiveness and Cough Peak Flow

Cough strength and effectiveness can be objectively assessed using CPF, typically in cooperative children aged ≥6 years. CPF is a practical and non-invasive technique assessed via a forceful cough following deep inspiration through a mouthpiece or mask connected to a peak flow meter [35,38,49].

In healthy adults, CPF typically exceeds 400 L/min. In contrast, values below 270 L/min in adults and likely in children over 12 years of age are associated with an increased risk of secretion retention and respiratory failure, even during otherwise mild RTIs. CPF values below 160 L/min are considered insufficient for effective airway clearance [38,44]. In children under 12 years of age, a CPF below the 5th percentile for age is similarly considered indicative of impaired cough effectiveness and heightened vulnerability to respiratory complications, underscoring the importance of age-specific reference values when evaluating cough function in pediatric populations [44,50,51].

A population-based study involving 649 healthy children and adolescents aged 4–18 years established such reference values, demonstrating that CPF is significantly associated with gender, height, and body surface area (*p* < 0.001). In contrast, age, though correlated, did not independently improve predictive accuracy. CPF values were generally higher in males than in females across all age groups, with fiftieth percentiles ranging from 147 to 488 L/min in females and 162 to 728 L/min in males. These findings support the clinical relevance of using age- and sex-specific CPF percentiles when assessing cough strength and identifying children at increased risk of respiratory complications [50].

Further evidence from a retrospective study of 366 pediatric patients with NMDs highlighted the need for pediatric-specific CPF reference values in this population. The study found that mean CPF values were significantly lower in patients with DMD (255.8 L/min) and congenital muscular dystrophy (249.1 L/min) compared to those with CMT disease (321.5 L/min), with *p*-values of 0.007 and 0.020, respectively. Additionally, children under 10 years of age had markedly lower CPF values than older patients (179.5 L/min vs. 300.9 L/min, *p* < 0.000). These findings highlight that adult-derived CPF thresholds may not be appropriate for pediatric NMDs and that tailored reference values are essential for guiding the initiation of assisted cough interventions in this group [51].

Traditionally, CPF and spirometry—including peak expiratory flow (PEF)—are measured using separate devices, increasing clinical workload and operational costs. Recently, a retrospective study including 40 children (median age 14.95 years) with NMDs who completed reproducible CPF and spirometry testing on the same machine (either Vyaire Body Box™ or Vyaire Pneumotachograph™, Höchberg, Germany) was conducted to assess the correlation between CPF and PEF. Median PEF and CPF were 4.05 L/min and 4.29 L/min, respectively. The analysis revealed a strong correlation between PEF and CPF (*p* = 0.03), with a coefficient of determination (R^2^) of 0.93. This suggests that more than 90% of the variability in CPF can be predicted by PEF values. Therefore, in children with NMDs, PEF measured by spirometry may serve as a useful estimate of CPF. However, for patients who are unable to perform valid spirometric manoeuvers, direct CPF measurement remains a valuable and informative alternative [52].

Cough Volume Acceleration (CVA) could represent another key parameter for evaluating cough effectiveness in children with NMDs. It is defined as the ratio of CPF to the PEF rise time and it reflects the dynamic capability of the cough to generate sufficient shearing forces for effective airway clearance [20]. To date, studies investigating CVA have predominantly focused on adult populations, particularly individuals with amyotrophic lateral sclerosis. In this population, CVA has been associated with aspiration and has been used to detect penetration and aspiration [53,54,55]. However, data on pediatric populations with NMDs remain lacking.

#### 3.2.4. Tests of Respiratory Muscle Strength

Diagnostic tests have been developed to assess selective weakness in the inspiratory, expiratory, and bulbar muscle groups. Commonly used measures include maximum inspiratory pressure (MIP), MEP, and sniff nasal inspiratory pressure (SNIP), which provide non-invasive estimates of respiratory muscle strength and may serve as indirect indicators of cough effectiveness [20,38,44].

MIP is defined as the average pressure sustained over one second when the patient inhales deeply against an occluded mouthpiece from residual volume, also known as the Mueller manoeuvre, while MEP is the average pressure sustained over one second when the subject exhales forcefully against an occluded mouthpiece from total lung capacity (TLC), known as the Valsalva maneuver. MEP ≥ 60 cmH_2_O is required to generate an effective cough, whereas MEP ≤ 45 cmH_2_O indicates impaired expiratory strength. Finally, SNIP is measured using a plug inserted into one nostril and connected to a manometer while the subject performs a sharp, voluntary inspiratory manoeuvre (a “sniff”) through the nostril. This maneuver is initiated from functional residual capacity (FRC). Although SNIP may be easier to perform and more suitable for patients with facial muscle weakness, it may require over 10 attempts for consistent results [20,38].

In a cross-sectional observational study of 42 DMD patients (mean age of 16.1 ± 4.0 years), both MIP and MEP showed significant correlations with CPF (*p* < 0.01), with MEP showing the strongest association (*p* = 0.051). These findings highlight expiratory muscle weakness as a primary factor in cough inefficacy, suggesting the importance of assessing and targeting expiratory muscle strength in the management and rehabilitation of these subjects [56].

## 4. Care Strategies for Managing Cough Insufficiency

**Figure 3:**
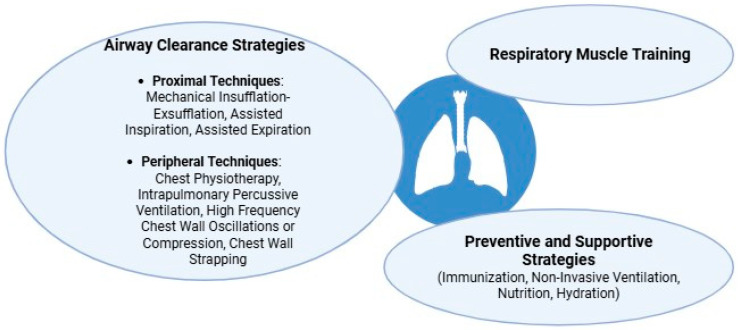
Main strategies for managing cough insufficiency in pediatric NMDs.

Cough insufficiency is a major contributor to respiratory morbidity in children with NMDs, requiring targeted care strategies to optimize airway clearance, prevent pulmonary complications, and preserve long-term respiratory function (Figure 3) [57].

### 4.1. Airway Clearance Techniques

Effective airway clearance remains a significant clinical challenge in managing impaired cough among children with NMDs [58]. Conventional independent techniques—such as deep inspiration followed by forced expiration—are often inadequate due to impaired respiratory muscle strength [38]. Similarly, oscillatory positive expiratory pressure devices may be poorly tolerated in very young or severely weak children, as they demand coordinated effort, a deep inhalation, and controlled exhalation against resistance [59]. Consequently, assisted airway clearance strategies are essential for improving secretion mobilization and reducing the risk of respiratory complications [38].

These techniques are generally classified as proximal or peripheral, based on the anatomical target of secretion mobilization. Proximal techniques aim to enhance cough efficacy and clear secretions from the central airways, whereas peripheral techniques focus on mobilizing secretions from the distal airways toward the larger bronchi [60].

#### 4.1.1. Proximal Airway Clearance Techniques

##### Mechanical Insufflation–Exsufflation

Mechanical Insufflation–Exsufflation (MI-E), or “cough assist”, is a cornerstone intervention for children with ineffective cough and respiratory muscle weakness [59,61]. The device simulates a natural cough by alternating positive pressure (to insufflate the lungs) with rapid negative pressure (to facilitate exsufflation), thereby generating high expiratory flow rates of 6–11 L/s [38,59,61].

MI-E is particularly beneficial in severely weak patients, having bulbar dysfunction, or unable to tolerate or perform assisted inspiration or expiration strategies. It can be delivered non-invasively via a mask or mouthpiece or invasively through a tracheostomy [38,58,60,62].

Clinical evidence from a multicenter retrospective review of 181 patients (including 51 children) who received MI-E between 2014 and 2018 offers insight into current practice. The device was typically prescribed to the most severely affected individuals, particularly those with a CPF below 160 L/min. Median insufflation and exsufflation pressures were 25 cmH_2_O and −35 cmH_2_O, with durations of 1.5 and 1.8 s, respectively. All patients used high inspiratory flow settings, with exsufflation pressures and times exceeding insufflation parameters (*p* < 0.001). CPF did not correlate with pressure settings, and adherence to daily use was highly variable, though highest among those with tracheostomies or SMA type I. The median duration of MI-E use was 17 months, and 96% of patients were concurrently on ventilatory support [63].

Additional evidence comes from a retrospective chart review of 98 intubated subjects with single-organ respiratory muscle failure who were unable to meet standard ventilator-weaning criteria. By using MIE with insufflation and exsufflation pressures of 60–70 cmH_2_O used via the endotracheal tube to normalize oxygen saturation (≥95% in ambient air) and facilitate airway clearance, 97 patients—including 41 children (26 with SMA type I and 15 with SMA types II and III)—were successfully extubated to continuous noninvasive ventilatory support (CNVS). Vital capacity (VC) increased by 270% (*p* < 0.001) from pre- to post-extubation, and among those who had not been CNVS-dependent prior to intubation, all were eventually weaned to part-time NVS. Only one patient required tracheostomy. These findings highlight the potential of MIE to normalize oxygenation, enhance VC, and allow successful extubation of patients otherwise considered “unweanable” [64].

Furthermore, a recent single-center retrospective study involving 37 children (median age 5.2 years) with NMDs demonstrated a significant reduction in RTIs requiring hospitalization after the introduction of daily MI-E. RTI-related admissions per 1000 eligible days declined from 3.7 to 0.9 (*p* = 0.006), and hospitalization days decreased from 33.6 to 2.7 (*p* = 0.001) [65].

Despite these encouraging outcomes, there is no standardized approach to MI-E settings in pediatric care. A European survey of 10 neuromuscular centers reported substantial variability in MI-E use across 240 children (mean age 10.5 years). Most centers (71%) used automatic mode; insufflation and exsufflation times ranged from 1 to 4 s, while pressure settings varied from 10 to 50 cmH_2_O (positive) and −10 to −60 cmH_2_O (negative). Notably, settings were frequently asymmetric and tended to increase with age (*p* < 0.001). These findings underscore the lack of clear pediatric guidelines and highlight the need for further research to establish age-appropriate, evidence-based MI-E protocols [66].

MI-E is generally well tolerated, although some children may experience thoracic wall discomfort, agitation, or crying during sessions. High device costs and limited reimbursement remain barriers to accessibility in certain healthcare systems [60].

##### Assisted Inspiration

Assisted inspiration techniques are divided into single-breath and breath-stacking manoeuvers [60].

In single-breath techniques, lung volume is augmented using a bag-valve mask, a noninvasive ventilator (in pressure-or volume-controlled mode), or an intermittent positive-pressure breathing (IPPB) device. A single deep insufflation is delivered via a mouthpiece or oronasal mask to approach lung insufflation capacity, followed by an unassisted or manually assisted cough (MAC) [38,60]. In a prospective study involving 29 children (aged 12.6 ± 3.6 years) with various NMDs, IPPB-assisted hyperinsufflation significantly increased FVC from 0.68 ± 0.40 L to a maximum insufflation capacity (MIC) of 1.05 ± 0.47 L (*p* < 0.001). Unassisted CPF improved in 27 of 29 patients, from 119.0 ± 57.7 L/min to 194.5 ± 74.9 L/min (*p* < 0.001). A moderate correlation was found between volume augmentation and CPF improvement (*p* < 0.05), supporting IPPB as an effective cough augmentation strategy in pediatric NMDs [67]. Limitations include financial cost and resource demands. When using bag-valve masks, size must be age-appropriate: approximately 220–360 mL for infants, 650 mL for children, and >1500 mL for adults, with volumes titrated accordingly [60].

Breath stacking, or stacked-breath assisted inspiration, involves repeated insufflations without exhalation until MIC is reached. Techniques include air stacking (active lung volume recruitment) using a bag-valve mask or a lung volume recruitment circuit equipped with a one-way valve or a volume-cycled ventilator. Glossopharyngeal breathing, also known as “frog breathing”, is another method, wherein air is pumped into the lungs using the oropharyngeal muscles to bypass inspiratory muscle weakness. Once the patient reaches near TLC, a cough is initiated, either spontaneously or with MAC [38,60,68,69]. Glottic function can limit the feasibility of some techniques, although one-way valves may mitigate this. If patients are unable to perform breath stacking, single assisted inspiration may be used as an alternative [60].

##### Assisted Expiration

Among assisted expiration strategies, MAC is the most widely used, involving a well-timed abdominal thrust or costophrenic compression synchronized with the patient’s cough effort. These manoeuvers increase intra-abdominal or intrathoracic pressure to enhance expiratory airflow and facilitate secretion clearance, typically following a spontaneous or assisted inspiration. The direction of compression is critical and should align with natural chest wall motion, except in Heimlich-type techniques where the thrust is directed upward and inward. While effective, MAC requires patient cooperation and precise caregiver coordination. Limitations include reduced effectiveness in patients with severe scoliosis, contraindications shortly after meals, and increased risk of rib injury in those with osteoporosis [60].

Exsufflation-only techniques, delivered via a MI-E device using negative pressure without prior insufflation, aim to replicate the expulsive phase of a cough. Although potentially beneficial, clinical data supporting this approach remain limited, and high device costs may restrict its availability in certain settings [60].

#### 4.1.2. Peripheral Airway Clearance Techniques

Peripheral techniques include manual chest physiotherapy (e.g., percussion, vibrations and shaking), intrapulmonary percussive ventilation (IPV), high frequency chest wall oscillations (HFCWO), high-frequency chest wall compression (HFCWC), and chest wall strapping (CWS) [60].

IPV, a modified form of IPPB, delivers high-frequency bursts of air (200–300 cycles per minute) at peak pressures ranging from 20 to 40 cmH_2_O synchronized with the patient’s breathing pattern. These internal percussions aid secretion mobilization and can be an alternative or adjunct to conventional manual techniques [38,60,70]. Evidence suggests that IPV may offer significant clinical benefits in children with NMDs. In a RCT of 18 adolescents (11–19 years) with NMDs, IPV significantly reduced the number of antibiotic days (0 vs. 24/1000 patient-days; IRR: 43, 95% CI: 6–333) and hospitalization days (0 vs. 4.4/1000 patient-days; IRR: 8.5, 95% CI: 1.1–67) compared to incentive spirometry. No episodes of pneumonia or bacterial bronchitis occurred in the IPV group, suggesting its preventive value despite statistical limitations [71]. IPV’s limitations include high cost, lack of standardized settings, and the need for specialized training. In pediatric patients, uncontrolled use may lead to hyperventilation, necessitating careful monitoring of arterial CO_2_ levels [60].

HFCWO is a non-invasive modality that employs a snug-fitting inflatable vest or cuirass to deliver rapid oscillatory chest wall compressions, typically at 5–20 Hz frequencies. These oscillations induce airflow changes that mobilize secretions from peripheral to central airways, facilitating clearance [38,60,72]. While widely used in various respiratory conditions, the evidence supporting HFCWO in NMDs is limited, and clinical response in pediatric populations can be variable. It is typically reserved for cases where more active airway clearance strategies such as MI-E are not tolerated [73]. Given its potential to mobilize substantial volumes of mucus, suction equipment and resuscitation devices should be readily accessible during use [38].

HFCWC delivers intermittent positive air pulses into an inflatable jacket, generating chest wall compressions at 5 and 20 Hz frequencies. This oscillatory action increases airway airflow, mobilizing secretions from the peripheral to central airways, and can be used alongside ventilatory support [60].

CWS involves wrapping the thorax with elastic material to restrict chest wall motion, passively reducing FRC. This decreases pulmonary compliance, may increase the work of breathing and cause dyspnea, thus CWS is typically employed in conjunction with ventilatory support to mitigate these risks [60].

### 4.2. Respiratory Muscle Training

Respiratory muscle training (RMT) has been shown to improve respiratory muscle strength and endurance in healthy individuals [74]. These findings have led to its application in various clinical populations, including children with NMDs, to enhance both daytime and nocturnal ventilatory capacity, reduce respiratory exacerbations, and improve cough efficiency [38].

Respiratory muscle strength is typically assessed by measuring MIP and MEP at the mouth. Endurance is commonly evaluated via maximum voluntary ventilation sustained over 10–15 s or by the duration a patient can breathe against a set inspiratory load. Two primary approaches to RMT are employed, specifically strength training, involving repeated maximal or near-maximal static inspiratory and/or expiratory efforts against resistance (e.g., closed glottis or occluded valve), and endurance training, consisting of breathing against a variable resistor set to a fixed percentage of the individual’s maximal capacity, sustained for a defined period. Training regimens typically involve 10–20 min of exercise once or twice daily [38].

Among the various RMT approaches, inspiratory muscle training (IMT) has been particularly studied as a non-invasive intervention to counter respiratory decline in children with NMDs. A randomized crossover study evaluated the safety and efficacy of a 3-month IMT program in 23 children (median age 12.3 years), most of whom were non-ambulant (*n* = 14). Participants performed 30 breaths at 30% of their MIP using an electronic threshold-loading device twice daily. No training was conducted during the control period. The intervention was well tolerated, with no reported adverse events. Although IMT did not significantly affect hospitalization rates or the frequency of RTIs (*p* = 0.60 and *p* = 0.21, respectively), it led to significant improvements in respiratory muscle strength and cough efficacy. Specifically, MIP increased by a mean ± SD of 14.6 ± 15.7 cmH_2_O during the IMT period, compared to 3.0 ± 11.9 cmH_2_O during the control period (*p* = 0.010). CPF improved by 32.3 ± 36.6 L/min with IMT, whereas it declined by −16.6 ± 48.3 L/min in the control phase (*p* < 0.001). These findings indicate that a structured IMT program is safe, feasible, and may serve as an effective adjunct to enhance inspiratory muscle strength and cough performance in children with NMDs [75].

### 4.3. Preventive and Supportive Strategies

Prevention of respiratory infections represents a fundamental aspect of respiratory management, particularly as disease progression and age-related changes increase the risk of respiratory complications during adolescence and transition to adulthood [8].

Immunization constitutes a fundamental component of RTIs prophylaxis in this population. Beyond lowering the incidence of infection-related morbidity, vaccination contributes preserving pulmonary function by mitigating acute exacerbations that may otherwise precipitate respiratory decline. Inactivated vaccines, including seasonal influenza and pneumococcal vaccines, are particularly important, as they substantially reduce the incidence and severity of viral RTIs and associated complications, such as secondary bacterial pneumonia and respiratory failure, which are common in children with impaired cough and respiratory muscle weakness [76,77].

Currently, evidence addressing the prophylactic use of azithromycin in pediatric NMDs populations remains limited. Due to its dual role as both an antimicrobial and an immunomodulatory agent, azithromycin has demonstrated efficacy in reducing the frequency of pulmonary exacerbations in chronic lung diseases such as non-cystic fibrosis bronchiectasis, with observed benefits attributed to its anti-inflammatory effects on the airway epithelium and neutrophil function [78]. While extrapolation from other chronic pulmonary diseases suggests potential utility, caution is warranted in children with NMDs, particularly in those conditions where azithromycin has been associated with symptom exacerbation. Additionally, concerns about antimicrobial resistance and cardiac side effects, including QT prolongation, necessitate a careful risk-benefit assessment [79]. Further research is needed to define optimal protocols, safety, and long-term outcomes in this vulnerable population.

Noninvasive respiratory support also plays a critical role in managing nocturnal and daytime hypoventilation, and in reducing the frequency of RTIs and hospital admissions, while potentially preventing chest wall deformities and improving long-term respiratory outcomes [38,80,81].

Finally, nutritional and hydration support are critically important in maintaining cough efficacy. Malnutrition, which is common in children with advanced NMDs due to feeding difficulties and increased metabolic demands, weakens both inspiratory and expiratory muscles, thereby compromising cough strength. Adequate hydration is equally important, as it helps maintain mucus viscosity and supports more effective airway clearance. Thickened, dehydrated secretions are more difficult to mobilize, especially in children with already weak expiratory flow, leading to secretion retention and infection risk [38,82,83,84].

## 5. Conclusions

Effective cough management is a cornerstone of respiratory care in children with NMDs, as impaired airway clearance is a major contributor to pulmonary morbidity and mortality in this population. The early identification of respiratory involvement—often preceding overt clinical symptoms—is critical for initiating timely interventions and preventing complications. The objective assessment of cough strength, particularly through measures such as CPF, should be integrated into routine evaluations alongside regular PFT. These tools enable the development of personalized airway clearance strategies and support a proactive, rather than reactive, approach to care. A multidisciplinary care model—engaging pulmonologists, neurologists, and rehabilitation specialists such as physiotherapists and speech therapists—is essential to preserving respiratory function, minimizing hospital admissions, and improving clinical outcomes and QoL. Anticipatory, coordinated management mitigates the risk of respiratory deterioration and contributes to improved long-term prognosis.

Nevertheless, this review has some limitations. The available literature remains limited, and the heterogeneity in study designs and methodologies complicates direct comparisons of findings. In addition, some divergence in clinical practice across centers further limits the generalizability of recommendations. These factors underscore the need for future research and the establishment of standardized, evidence-based protocols to optimize respiratory care in this vulnerable pediatric population.

## Data Availability

No new data were created or analyzed in this study.

## Abbreviations

The following abbreviations are used in this manuscript:

CMT	Charcot–Marie–Tooth
CNVS	Continuous Noninvasive Ventilatory Support
CPF	Cough Peak Flow
CVA	Cough Volume Acceleration
CWS	Chest Wall Strapping
DMD	Duchenne Muscular Dystrophy
FEV_1_	Forced Expiratory Volume in the 1st second
FRC	Functional Residual Capacity
FVC	Forced Vital Capacity
HFCWC	High-Frequency Chest Wall Compression
HFCWO	High-Frequency Chest Wall Oscillations
IL	Interleukin
IMT	Inspiratory Muscle Training
IPPB	Intermittent Positive Pressure Breathing
IPV	Intrapulmonary Percussive Ventilation
MAC	Manually Assisted Cough
MEP	Maximal Expiratory Pressure
MIC	Maximum Insufflation Capacity
MI-E	Mechanical Insufflation–Exsufflation
MIP	Maximum Inspiratory Pressure
MUC	Mucin
NMDs	Neuromuscular Disorders
PEF	Peak Expiratory Flow
PFT	Pulmonary Function Testing
QMT	Quantitative Muscle Testing
QoL	Quality of Life
RCTs	Randomized Controlled Trials
RMT	Respiratory Muscle Training
RTIs	Respiratory Tract Infections
SD	Standard Deviation
SVC	Slow Vital Capacity
SMA	Spinal Muscular Atrophy
SNIP	Sniff Nasal Inspiratory Pressure
TLC	Total Lung Capacity
TRP	Transient Receptor Potential
TRPA1	TRP Ankyrin 1
TRPM8	TRP Melastatin 8
TRPV	TRP Vanilloid
VC	Vital Capacity
